# Pollutants and Their Interaction with Diseases of Social Hymenoptera

**DOI:** 10.3390/insects11030153

**Published:** 2020-03-01

**Authors:** Heike Feldhaar, Oliver Otti

**Affiliations:** Animal Population Ecology, Animal Ecology I, Bayreuth Center of Ecology and Environmental Research (BayCEER), University of Bayreuth, Universitätsstr. 30, 95447 Bayreuth, Germany; oliver.otti@uni-bayreuth.de

**Keywords:** disease susceptibility, fine particulate matter, heavy metal, pesticide, social insect

## Abstract

Many insect species, including social insects, are currently declining in abundance and diversity. Pollutants such as pesticides, heavy metals, or airborne fine particulate matter from agricultural and industrial sources are among the factors driving this decline. While these pollutants can have direct detrimental effects, they can also result in negative interactive effects when social insects are simultaneously exposed to multiple stressors. For example, sublethal effects of pollutants can increase the disease susceptibility of social insects, and thereby jeopardize their survival. Here we review how pesticides, heavy metals, or airborne fine particulate matter interact with social insect physiology and especially the insects’ immune system. We then give an overview of the current knowledge of the interactive effects of these pollutants with pathogens or parasites. While the effects of pesticide exposure on social insects and their interactions with pathogens have been relatively well studied, the effects of other pollutants, such as heavy metals in soil or fine particulate matter from combustion, vehicular transport, agriculture, and coal mining are still largely unknown. We therefore provide an overview of urgently needed knowledge in order to mitigate the decline of social insects.

## 1. Introduction

Currently, insect abundance and diversity are in decline worldwide [1,2,3]. Various factors are contributing to this decline [1,3]. Besides biological factors, habitat destruction, and climate change, one of the main drivers is anthropogenic pollution [1,3,4,5,6,7]. Anthropogenic pollutants such as pesticides, heavy metals, or airborne fine particulate matter originating from agricultural or industrial sources may have lethal or sublethal toxic effects on insects [1,3,4,7]. Sublethal health effects can translate into more dramatic effects, for example by increasing disease susceptibility [4,7,8,9,10,11,12,13] or decreasing tolerance towards other stressors, such as land use intensification [14,15,16,17]. In addition, such pollutants are known to negatively affect learning abilities and/or lower activity levels [18,19,20,21,22,23,24], which may compromise insects even further [19,21,25].

Not surprisingly, insect decline also affects social insects, including important pollinators such as wild social bees and honey bees [6,26,27]. Social Hymenoptera, such as bees, ants, and wasps, are characterized by the presence of overlapping generations within the colony, brood care, and reproductive division of labor, whereby, in most cases, a single or only a few female individuals reproduce, i.e., the queen(s) and other females, help to raise the brood as workers [28].

In social Hymenoptera, the effects of pollutants can therefore manifest on the level of the individual, as well as the colony. Individual foraging workers confronted with pollutants in their environment take up those pollutants into their bodies (at least into their gut), but also distribute them within the colony during food transfer. Especially larvae are helpless and need to be fed by adult workers. The lifespans of the workers are usually short in comparison to that of the queen and the colony as a whole, with its perennial life cycle, as with, e.g., in honey bees or ants. The sublethal effects of pollutants may be hardly measurable in individuals. However, these subtle individual-level effects may be amplified over time, resulting in long-term negative effects on colony fitness [19,29,30]. For example, pollutants incorporated into stored foods or nest materials, including beeswax, may accumulate over time [31,32,33,34,35,36,37,38,39,40], leading to a constant exposure to pollutants of larva and adults of new generations of workers, as well as sexuals [37,41,42]. In this way, even subtle effects on individuals can have quite extreme negative consequences for social insect colonies, threatening their existence [21,29,30,43]. 

As social Hymenoptera are central place foragers that constantly exploit resources in the surroundings of their nest, high local levels of pollutants can result in chronic exposure. Foraging ranges of social Hymenoptera such as honey bees or bumblebees tend to be significantly larger in comparison to those of solitary bees or solitary wasps that provision their offspring [44,45]. If polluted food patches can be avoided by social bees, this may make them less vulnerable to pollutants. Due to their considerably shorter foraging distances, we expect solitary Hymenoptera [46,47] to be more prone to pollution. This is especially so in ants, where foraging distances are considerably shorter, as only food sources within walking distance can be exploited. Variation in life history strategies among honeybees, bumblebees, and solitary bees might be another factor defining the strength of the effects induced by pollutants. While in honeybees, food may be detoxified before it is given to the queen, bumblebee queens and solitary bees come into contact with pollutants directly, since they go foraging themselves [48]. In addition, social species have a broader resource use and may be able to avoid particularly polluted food sources [49].

Thus, effects are likely to vary among taxa due to differences in foraging range, foraging mode, and type of food a species collects. The same aspects determining the encounter rate of pollutants might also define host-pathogen interactions and encounter rate with pathogens [50,51,52,53]. Foraging flights of bees and wasps are far more energetically demanding than foraging on foot [54]. However, for wasps and ants that feed on a higher trophic level than social bees, the bioaccumulation of pollutants along the food chain might an important factor compromising their health [36]. Consequently, the degree of exposure to pollutants is, on one hand, strongly linked to life history and nutritional ecology of social Hymenoptera, and will, on the other hand, affect the energetic requirements and metabolic rate of individuals and the colony as a whole.

## 2. Major Classes of Pollutants Threatening Social Insects

**Figure 1:**
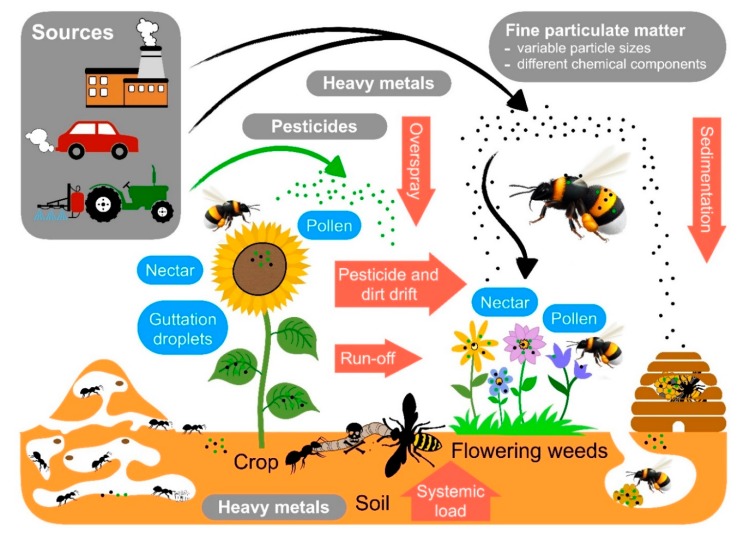
Sources of the environmental pollutants and exposure pathways of social insects to pollutants. Pesticides (including insecticides) derive mostly from agricultural sources, while heavy metals are released into the environment through industrial processes, combustion, or traffic. Fine particulate matter includes both, pesticides (or their residues) and heavy metals bound to particles of 10 µm and smaller. Fine particulate matter is composed of many different potentially toxic chemical components. Social insects can take up pollutants orally during foraging and then transfer them to the brood or incorporate them into nest material. In bees, pollutants can also end up in stored food, such as honey or bee bread. In addition, pollutants may be deposited on the cuticles of insects and their nests directly via the air. Subsequently, these may again be incorporated into nest material or even enter the insect’s body, e.g., via the tracheal system.

**Figure 2:**
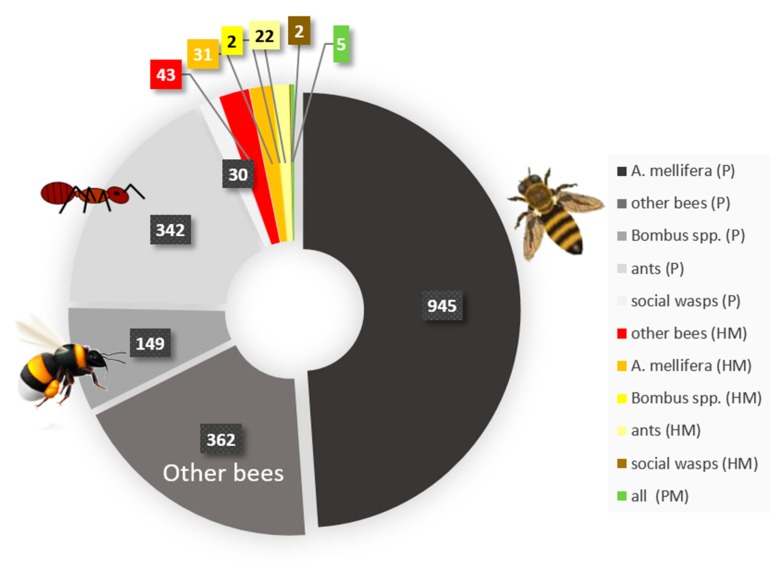
The proportion of publications that studied the different types of pollutants in relation to the different groups of social Hymenoptera on ISI Web of Science. Most studies investigated the effects of pesticides (P) on social Hymenoptera (n = 1828 in total; shaded in grey). Studies reporting the occurrence of pesticides in individuals, their food, or the nest were also included. Almost 75% of the studies were conducted on bees, most likely due to their importance as pollinators. Studies on ants in relation to pesticides show a rather narrow focus on the eradication of invasive species using insecticides. In total, only 98 studies elucidated the impact of heavy metals (HM) or the heavy metal content of bee products or nest material of social insects. Even fewer studies (n = 5) exist on the presence of particulate matter (PM) on the cuticle or its potential impact on any social Hymenoptera species. Search terms were either “Apis”, “Bombus”, “bumblebee”, “bee”, “ant”, or “wasp” for the group of social Hymenoptera with “health”, “toxic*”, or “effect“ as a keyword for the impact of the pollutants, and either “pesticide*”, “fungicide*”, “insecticide*”, “heavy metal”, “particulate matter” or “air pollution” as search terms for fine particulate matter. The first (newest) 50 publications per search were screened for the proportion of publications that did not fit thematically, and this percentage was subtracted from the original number of publications returned by the search.

Notwithstanding the worldwide dramatic decline in the abundance and species richness of insects [1,2,3], massive losses in honey bee colonies and the potential decline in wild bees as pollinators have caught the attention of scientists and the public alike [6,55,56,57,58]. Environmental pollution is regarded as one of the major drivers of (social) insect decline. Among the pollutants, pesticides used in agriculture have gained the most attention because pollinators visit crop plants or wild flowers growing near arable fields and are confronted with those chemicals when collecting food (Figure 1) [59,60,61,62]. Heavy metals are a second group of pollutants that may threaten social insects in agricultural regions, but also in more urbanized or industrial areas [31,35,63,64,65,66,67,68,69,70,71]. Heavy metals are present in soils (either naturally or due to pollution) and can potentially lead to contamination of the nests of social insects, or they may move up the food chain (Figure 2). Plants may take up heavy metals which, in turn, are ingested by social bees collecting pollen and nectar. Also, other herbivorous insects serving as prey for social wasps may accumulate these pollutants, which may result in the increased exposure of these predatory wasps to heavy metals [35,36,47,63,65,68,69,70,71,72,73,74,75,76,77,78,79,80]. Pesticides (or their residues) and heavy metals, as well as other pollutants from combustion, traffic, agriculture, and coal mining, can also form or bind to fine particulate matter [66,81,82,83,84,85,86,87,88,89,90,91] that pollutes the air. Eventually, fine particulate matter settles on surfaces, such as insect cuticles [88,89] or flowers [92] (Figure 2). From there, social bees might collect these particles, together with nectar or pollen. Fine particulate matter is defined by the size of particles, rather than the specific substance class of which it is composed [93,94,95]. It may be composed of different chemical components, such as ammonium, silicon, sulfate, nitrate, elemental carbon matter, organic carbon matter, sodium, heavy metals, or phenolic compounds [93,96]. This is unfortunate, because we think that the detrimental effects on social insect health is likely to be mediated by both, i.e., small particles and their chemical properties [97,98]. Particle size potentially determines whether particles can enter tissues, whereas the chemical properties potentially define the degree of toxicity or reactivity with tissue.

The effect of pesticide exposure on social insects has so far received far more attention than heavy metals or fine particulate matter (Figure 2). In honeybees and wild bees (including bumblebees), the detrimental effects of pesticides and insecticides have been studied as non-target effects on these important pollinators, and thus, in the light of conservation. In addition, the accumulation of pesticides (and to a lesser extent, heavy metals) in beeswax and honey has received much attention [31,37,38,40,70,99,100,101,102,103,104,105,106,107]. Since pollutants may reach the human consumer via these bee products, they may be used for biomonitoring of the environment, and most importantly, may mediate detrimental effects upon offspring, resulting in colony-level detrimental effects. In contrast to social bees, ants and wasps are often regarded as targets of insecticides, e.g., in the eradication programs of invasive species [108,109,110]; however, native ant and wasp species may also suffer from such eradication programs as non-target organisms [111,112]. Abundant evidence exists from laboratory studies, but also field studies, that pesticides have detrimental health effects on social bees at levels encountered in the environment (see [113,114] for excellent overviews on the impacts of systemic insecticides on social insects and organisms in general). Several authors have found a positive correlation between the pesticide levels found either in bees themselves or pollen and the mortality rates of individuals or number of workers/colony development in the field [115,116,117,118,119,120]. Risk assessments correlating pesticide residues found in pollen or honey with health effects also strongly support that many bees in agricultural landscapes are threatened by the pesticide levels they encounter under natural conditions [34,114].

In comparison to pesticide effects, the health effects of heavy metals have received much less attention (Figure 2). Of the existing studies, many have established that heavy metals are often present in the nest matrices in ants and the wax combs or honey in bees in polluted areas [31,35,63,65,67,69,70,71,75,78,102,121,122,123,124,125,126,127,128,129,130]. While many social Hymenoptera may be confronted with heavy metal pollution in strongly human-influenced environments, relatively little data exists on the potential detrimental health effects in comparison to pesticide effects. The detrimental effects of heavy metals include sublethal effects such as impaired learning and memory, as well as higher mortality correlating with higher levels of heavy metal pollution in studies using pollution gradients [46,47,64,71,131,132,133,134,135,136,137,138,139].

The potential detrimental effects due to fine particulate matter have hardly been studied at all. Again, as for bee products, it is suggested that honeybees could be used for biomonitoring, since they collect particles on the cuticle [88,89]. Both heavy metal contamination and fine particulate matter have been shown to impact human health [140,141]. Therefore, we expect these stressors to also harm insects, including social insects [73,136].

## 3. Pathogens of Social Insects

Social insects are confronted with a plethora of pathogens that include viruses, bacteria, fungi (including the microsporidian bee parasite *Nosema* spp. or entomopathogenic fungi entering through the cuticle [142,143,144]), as well as protozoa (such as the widespread gut trypanosome *Crithidia* in bumblebees) and metazoan parasites and parasitoids [52]. Here we only provide a very short overview over the pathogens and parasites mentioned in this review.

Due to the commercial importance of bees and the long tradition of bee keeping, honeybee pathogens are the best studied pathogens. With recent advances in sequencing techniques, many viruses of social Hymenoptera have been characterized [50,51,145], with some being well known due to the phenotypes they induce in infected individuals, such as deformed wing virus (DWV), that is widespread among honeybees as well as bumblebees [50,146]. In all groups of social Hymenoptera, viruses may have a high prevalence within a species. Viruses are often transmitted horizontally among individuals and between species or genera. Depending on the host–virus pair, the health effects can range from asymptomatic to symptomatic infections, with individuals suffering from altered behavior, higher mortality, or morphological deformations [147,148,149,150], and some viruses have been shown to have higher virulence in stressed individuals [51,151]. Both, the microsporidian *Nosema* spp. and the trypanosome *Crithidia bombi* are common parasites in bumblebees, of which the latter can reach high prevalence within populations [152]. *Crithidia bombi* is a generally benign gut parasite taken up during foraging. However, it has been shown to impact individual and colony level fitness by impairing foraging ability, decreasing worker longevity, or reducing colony founding success [153,154,155]. *Nosema* spp. are intracellular microsporidian parasites that are ingested and then infect cells in the gut. From the gut, an infection (of at least *N. ceranae*) can spread to other tissues within the body. Similar to *Crithidia* spp., the fitness effects of *Nosema* infection can be mild. However, the induced damage to the midgut tissue by the parasite can result in higher mortality rates and colony failure [156,157]. Like in viruses, the virulence of *Nosema* spp. and *Crithidia* spp. may be context-dependent, and detrimental effects on hosts can be stronger when multiple stressors interact [153,158].

Below, we review the interactive effects of pathogens (if known) with groups of pollutants that social insects are often confronted with, pesticides whose residues are often ingested by foragers in agricultural landscapes, and heavy metals that may occur in soil. Both pesticide residues and traces of heavy metals, among other compounds, make fine particulate matter, for which studies on the effects on animals other than humans are lacking. We review how different groups of pollutants such as pesticides, heavy metals, and fine particulate matter affect social insects in relation to physiology and immune reactions.

## 4. Pollutants and Disease Susceptibility of Social Insects—Mechanisms of Interaction

The insect immune system is generally comprised of three interlinked parts: (1) the cuticle and other epithelia that act as a barrier towards potential pathogens and other stressors from the environment, (2) the humoral immune response, and (3) the cellular immune response [159]. In social insects, interactions among individuals and/or the treatment of the nest and stored foods with immune effectors or venom adds a fourth component to the immune response [160,161].

The cuticle itself, as well as other epithelia beneath the cuticle or lining the trachea or alimentary tract, act as a physical and chemical barrier. Some pollutants, such as fine particulate matter might be effectively stopped by this barrier and accumulate on the cuticle [88]. Pathogens or tissue damage elicit a local immune response, leading to the production of antimicrobial peptides (AMPs) or reactive oxygen species (ROS). In addition, the humoral arm of the insect immune system includes pattern recognition proteins that identify invading microbes or other internalized non-self objects. Upon recognition of microbes, the synthesis of antimicrobial effector proteins such as AMPs, but also serine proteases, is initiated and regulated mainly via different signaling pathways, i.e., Toll, Imd, and Jak-STAT [162]. Upon recognition, serine proteases trigger the prophenoloxidase cascade, which results in melanization reactions, important in wound healing and parasite and non-self particle encapsulation. The cellular arm of the insect immune system consists of pathogen recognition and the subsequent phagocytosis, nodulation, or encapsulation of invading microbes, parasites, or non-self objects involving hemocytes, as well as the production of melanin and melanization reactions and the production of ROS [159]. We expect the phenoloxidase cascade to be able to encapsulate and melanize particles of pollutants, because early studies in ecological immunology have shown that nylon filaments and sephadex beads are successfully encapsulated and melanized by the PO cascade [163,164,165]. In addition to these interlinked components of the immune system, insects possess detoxification mechanisms that have evolved to prevent damage from environmental toxins such as plant secondary compounds or toxins produced by fungi or bacteria. Such detoxification mechanisms also play a role in metabolic resistance towards synthetic toxins and pollutants. The main enzyme superfamilies involved in detoxification in insects are cytochrome P450 monooxygenases (P450s), carboxylesterases (COEs), and glutathione –S-transferases (GSTs) [166,167].

Pollutants may impair the health of social insects and thereby enhance disease susceptibility via several different mechanisms that are directly or indirectly related to the immune system. First, pollutants may interfere directly with the immune system and thereby have an immunosuppressive effect. Insecticides with neurotoxic effects have been shown to modulate signaling pathways of the immune system [10] as well as hemocyte number [12], promoting pathogen replication or resulting in higher mortality of pathogen-exposed honey bees. The expression of AMPs may also be influenced, although results are ambiguous, and data on AMP-levels in the hemolymph are largely lacking [4,168]

Second, pollutants may interfere either with detoxification mechanisms within the insect’s body or the ability of social insects to sterilize stored food sources [169,170,171]. An increase in the production of ROS in the midgut after the oral uptake of insecticides can lead to oxidative stress and disruption of the oxidative balance in the gut [172], which may weaken the midgut’s barrier function towards pathogens. The fungicide iprodione, but also heavy metals, have been shown to damage the midgut epithelium, which may, on one hand, result in a decrease immunocompetence of the tissue, and on the other, in a decrease in metabolic activity and decreased energetic efficiency due to mitochondrial damage [80,173].

Third, pollutant exposure may affect social insect cognitive abilities [131,132,174], which often translates into reduced individual or colony-level fitness due to behavioral changes with potential knock-on effects on disease susceptibility. Immune competence has been shown to be compromised in starved social insects [153,175], stressing the energetic costs involved in mounting an immune response [176]. Thus, reduced foraging performance due to stress-induced impaired cognitive abilities may trigger a cascade of food shortage when lower quantities or quality of resources are collected [20,174,177], or alter the social networks of nursing individuals and impair thermoregulation [43]. The disruption of social networks may also translate into altered grooming behavior, which may increase the susceptibility to pathogens breaking through the barrier of the cuticle, such as fungal entomopathogens, as spores are no longer removed from a nestmate´s cuticle [178].

Last, symbionts of social insects such as the gut microbiota that play an important role in host health [179,180,181] may be affected by pollutants. Pesticides and heavy metals have been shown to induce changes in the composition of the microbiome in honey bees, which may impact host physiology [182,183,184,185,186].

## 5. Pollutants Commonly Encountered by Social Insects and Their Interaction with Disease Susceptibility

Social insects are ubiquitous in terrestrial ecosystems, and also occur in highly human-altered and/or polluted landscapes such as agricultural landscapes, urban areas, or sites heavily influenced by industry [88,133,187,188,189]. Their existence in many different habitats and their colony lifestyle make them ideal study systems, because different degrees of pollution and different pollutants can be studied in the same species and in individuals with the same genetic background. In the environment, social insects may often be confronted with a mixture of pollutants and other environmental stressors including pathogens [6,50,55,190]. Therefore, most studies looking at the interactive effects of pollutants and pathogens have been conducted under laboratory conditions [4]. Controlled conditions facilitate a mechanistic understanding of how pollutants interact with pathogens, and physiological parameters have been widely measured. Different physiological pathways might be impaired directly or indirectly, leading to variations in interactions with pathogens. Under field conditions, the end point measured is usually mortality on the individual level or colony development.

## 6. Pollutants: Pesticides

Pesticides, such as insecticides and fungicides, are the most prominent groups of pesticides with which social insects are confronted [7]. Managed honeybees are often treated with acaricides when infested with the parasitic *Varroa*-mite [191,192]. In addition, during foraging, honeybee workers take up pesticide residues (Figure 1), leading to the accumulation of pesticide residues in stored food such as honey or bee bread [34,104,193,194,195].

Different groups of pesticides reduce the health of social insects in variable ways, and thereby, influence disease susceptibility via different routes. Most insecticides are neurotoxins, such as organophosphates or methylcarbamates that inhibit the acetlylcholinesterase, or neonicotinoids that are agonists of the nicotinic acetylcholine receptor [191]. Sublethal doses of these neurotoxins affect learning abilities and memory in honeybees and bumblebees, reducing individual foraging efficiency, navigation ability, motor function, and social behavior in the nest. The reduction in those traits negatively affects the nutritional status of colonies [18,24,25,43,196] and weakens them. The field foraging behavior of bumblebees was shown to be only mildly affected by pesticides, but exposed colonies have fewer adult workers and sexuals [19]. In bumblebee queens, neonicotinoid exposure reduces the probability of a colony being founded [197]. We predict that most of these conditions will indirectly increase disease susceptibility.

In honeybees and bumblebees, sublethal neonicotinoid exposure at concentrations comparable to those found in nectar and pollen affects the immune system negatively, resulting in a reduction of hemocyte density, encapsulation response, and antimicrobial activity [9,10,198,199]. Similar to vertebrates, exposure to neonicotinoids also induces oxidative stress by alteration in retinoid metabolism [200]. The neonicotinoid thiamethoxam alone already influences expression patterns of immune related genes in bee larvae as well as adults [201,202]. When adult bees were additionally confronted with the gut parasite *Nosema ceranae*, the mortality of adult bees increased, suggesting a synergistic effect of the pesticide with the pathogen [201].

Combinatorial effects of different insecticides have been shown to increase honeybee mortality [203], but the insecticides seem not to act synergistically. Chronic insecticide exposure suppresses immune-related genes and leads to stronger changes in immune gene expression in the gut than single insecticide exposure. However, neonicotinoids can increase parasite or pathogen susceptibility and enhance negative fitness effects [13,204]. While some studies did not find interactive effects between pesticides and pathogens [4], many did. After exposure to sublethal doses of pesticides, the mortality of honeybees was higher when infected with the gut parasite *Nosema ceranae* [8,169,203,205,206,207]. Pesticides can also alter susceptibility to viruses, thereby increasing mortality [171,208]. Such an increased impact of viruses may be mediated by the parasitic mite *Varroa* that often carries bee viruses, but neonicotinoids may also interact with *Varroa* to reduce honeybee survival without virus transmission [209]. Together with the effects on the immune system, this highlights the urgent need for more extensive investigations into the interaction between pesticides, other pollutants, and disease susceptibility.

## 7. Pollutants: Heavy Metals

Heavy metals released from industry as well as traffic and might enter the soil or remain airborne as fine particulate matter (e.g., heavy metals may be contained in particles of break wear; see below). From soil, heavy metals can be taken up by plants and get into food sources such as nectar or pollen, or may be airborne. Airborne fine particulate matter might sediment on flowers and leaves, from where it can be ingested by insects (Figure 1). The accumulation of heavy metals during foraging has been documented along pollution gradients. Pollution levels in pollen stores of bees were positively correlated with heavy metal pollution as well as the heavy metal content in the bodies of bees [46,71], ant workers or nest material [35,133], and wasps [36,80]. Correlative studies have shown that with increasing heavy metal pollution, the mortality of solitary [46,47] as well as social bees [64,210] increased, and that body size [35] and colony size decreased in ants [133].

Higher levels of heavy metals in the insect body lead to diverse physiological effects. Within the body, heavy metals seem to be sequestered as mineralized spherites into different tissues such as the intestinal tissue, fat body, or Malphigian tubules [80,211,212] as a detoxification mechanism. In the social wasp *Polistes dominula*, heavy metal contamination leads to alterations in the midgut tissue on the cellular level. This hints at damage of the mitochondria and the epithelial cells themselves, because the microvilli were disorganized [80]. Also, cell nuclei contained a higher amount and density of heterochromatin [80]. Honeybees fed with food contaminated with sublethal concentrations of heavy metals (Cadmium oxide (CdO) and lead oxide (PbO)) showed similar cellular damage in the midgut tissue and a disruption of the peritrophic membrane [213]. Damage of the peritrophic membrane, the midgut tissue, or an altered gut microbiome due to the uptake of heavy metal may increase the susceptibility of social insects to gut pathogens such as *Nosema* spp. or bacterial pathogens.

Aside from tissue damage, heavy metals have been shown to interfere with the immune system of social Hymenoptera. In *Formica aquilonia* ants, the encapsulation response was elevated at moderate levels of heavy metal contamination and suppressed at high levels. Such a reduction in the encapsulation response might make these ants more susceptible to infection [138], and may explain the smaller colony sizes in heavily polluted sites [133]. Similarly, feeding honeybee workers with sublethal concentrations of Cd significantly impaired the ability to prevent bacterial proliferation after an immune challenge with live *E. coli* 3 days after exposure [212].

Only very few studies exist which have investigated the interactive effects of heavy metal pollution and pathogens or parasites. Szentgyorgyi et al. [71] did not find a correlation between heavy metal contamination levels and the prevalence of the microsporidian parasite *Nosema bombi* in bumblebees. More research is warranted on the interplay of heavy metal pollution with disease susceptibility across a wide range of Hymenopteran taxa.

## 8. Airborne Fine Particulate Matter

In spite of fine particulate matter being a pervasive air pollutant, it has received relatively little attention vis-à-vis its health impacts on insects in general. While other pollutants are defined by their composition, these airborne solid and liquid suspensions are usually defined by the size of their particles. Especially particulate matter smaller than 10 µm and 2.5 µm in diameter (called PM_10_ or PM_2.5_ respectively) has been shown to have negative effects on human health, since it can enter respiratory tissue, the blood stream, or even the fetal side of the placenta, as recently shown [214], and induce, e.g., vascular dysfunction, systemic oxidative stress, and inflammatory events [215,216]. Airborne fine particulate matter derives from combustion or break wear in urban areas, agricultural dusts, coal mining, and other industries [217,218]. Its chemical composition strongly depends on the source of the particles [218]. While dust from coal mines is mostly comprised of carbon particles, car exhaust (especially diesel) usually comprises aggregates of a carbon core where organic residues and heavy metals are deposited. Brake wear particles may contain metallic components as well as phenolic compounds from brake pads. Fine particulate matter also comprises particles forming in the atmosphere through chemical reactions, such as through the oxidation volatile compounds such as sulfates or nitrates that form from the oxidation of sulfur dioxide or nitrogen dioxide. The toxicity of the particles depends on their size and chemical composition [218].

Insects are likely to ingest fine particulate matter via their food, as it may either be in the nectar or have settled on other surfaces such as plant material [86,87,91,92]. In social insects and especially honeybees, the collection of fine particulate matter during foraging translates into an accumulation of xenobiotics in honey and beebread. Comparisons between apiaries have often shown higher levels of residues of pesticides, heavy metals, and other particulate matter in the bees themselves or honey in urbanized or industrialized areas in comparison to rural areas [32,66,70,210,219,220].

To date, the effects of airborne fine particulate matter on insect health have barely been studied. While studies show that pathogen pressure (measured as the prevalence of a variety of bee pathogens) may be higher in urbanized areas, this has been discussed in the light of pathogen transmission and general epidemiology [190]. However, especially fine particulate matter levels have been shown to be elevated in urban areas [86], and honey bees have been shown to accumulate airborne particulate matter from a cement factory and traffic on their body surfaces in highly polluted areas [88,89]. Such particulate matter may thus contribute to enhanced disease susceptibility and mortality of social insects [66,69]. While this is correlative evidence that higher levels of airborne pollution may have negative effects on insect health, a few studies have attempted to uncover the effects more directly. Late-instar larvae of the cotton bollworm *Helicoverpa armigera* showed higher mortality rates when feeding on leaves laden with coal dust that was obtained by milling coal from a coal mine. In contrast to late-instar larvae that did not adjust their feeding behavior, early instars avoided feeding on coal dusted leaf material [91]. Artificial haze smoke (mimicking haze from forest fires) was also shown to increase mortality in lepidopteran larvae. Direct exposure to haze contributed to increased mortality in caterpillars, and both direct exposure as well as the ingestion of haze-exposed food plants led to increased larval developmental time and decreased pupal weight. As no particulate matter was found in the trachea of the insects, the authors concluded that toxic smoke gases and toxic food may be detrimental, rather than particulate matter [90].

## 9. Outlook and Knowledge Gaps

To date, studies on the interactive effects of pollutants with pathogens and disease susceptibility in social insects have concentrated on pesticides, while other important pollutants have mostly been studied in light of their direct detrimental effects. Again, here, heavy metals have received some attention, while airborne particulate matter—which has gained increasing attention due its impact on human health—remains understudied. Currently, we do not know whether differences in the physiological impact of pollutants and their interactions with pathogens depend on the mode of uptake. Most studies have administered pollutants with food. However, fine particulate matter may be taken up into the body via the tracheal system, and it may cross tissue boundaries such as the midgut tissue more easily due the small particle size. Under field conditions, different groups of pollutants will often interact, increasing the complexity. Likewise, they may be taken up via different exposure routes simultaneously. Therefore, to gain a mechanistic understanding of how pollutants interact with social insect physiology and their pathogens, controlled experiments under laboratory conditions with realistic concentrations of pollutants are required. Since the impact of pollutants and their interactive effects with pathogens will also strongly depend on other environmental conditions, comparative experiments in the field will also be vital. Lastly, a large part of the knowledge we currently have with respect to the range of pathogens present in social insect species and the health effects of pollutants is focused on very few commercially important social insect species (mostly *Apis mellifera* and *Bombus* spp.). We therefore need to undertake comparative work on similar pollutants and pollutant concentrations over a broader range of social insect species with different lifestyles and life histories, and compare the impacts of pollutants alone, as well as their interactive effects with pathogens.

## References

[1] Sanchez-Bayo F., Wyckhuys K.A.G. (2019). Worldwide decline of the entomofauna: A review of its drivers. Biol. Conserv..

[2] Hallmann C.A., Sorg M., Jongejans E., Siepel H., Hofland N., Schwan H., Stenmans W., Muller A., Sumser H., Horren T. (2017). More than 75 percent decline over 27 years in total flying insect biomass in protected areas. PLoS ONE.

[3] Wagner D.L. (2020). Insect declines in the anthropocene. Annu. Rev. Entomol..

[4] Collison E., Hird H., Cresswell J., Tyler C. (2016). Interactive effects of pesticide exposure and pathogen infection on bee health—A critical analysis. Biol. Rev..

[5] Goulson D. (2013). An overview of the environmental risks posed by neonicotinoid insecticides. J. Appl. Ecol..

[6] Goulson D., Nicholls E., Botias C., Rotheray E.L. (2015). Bee declines driven by combined stress from parasites, pesticides, and lack of flowers. Science.

[7] Sanchez-Bayo F., Goulson D., Pennacchio F., Nazzi F., Goka K., Desneux N. (2016). Are bee diseases linked to pesticides?—A brief review. Environ. Int..

[8] Aufauvre J., Biron D.G., Vidau C., Fontbonne R., Roudel M., Diogon M., Vigues B., Belzunces L.P., Delbac F., Blot N. (2012). Parasite-insecticide interactions: A case study of Nosema ceranae and fipronil synergy on honeybee. Sci. Rep..

[9] Czerwinski M.A., Sadd B. (2017). Detrimental interactions of neonicotinoid pesticide exposure and bumblebee immunity. J. Exp. Zool. Part A-Ecol. Integr. Physiol..

[10] Di Prisco G., Cavaliere V., Annoscia D., Varricchio P., Caprio E., Nazzi F., Gargiulo G., Pennacchio F. (2013). Neonicotinoid clothianidin adversely affects insect immunity and promotes replication of a viral pathogen in honey bees. Proc. Natl. Acad. Sci. USA.

[11] Fauser-Misslin A., Sadd B., Neumann P., Sandrock C. (2014). Influence of combined pesticide and parasite exposure on bumblebee colony traits in the laboratory. J. Appl. Ecol..

[12] Hernandez Lopez J., Krainer S., Engert A., Schuehly W., Riessberger-Gallé U., Crailsheim K. (2017). Sublethal pesticide doses negatively affect survival and the cellular responses in American foulbrood-infected honeybee larvae. Sci. Rep..

[13] O’Neal S.T., Anderson T.D., Wu-Smart J.Y. (2018). Interactions between pesticides and pathogen susceptibility in honey bees. Curr. Opin. Insect Sci..

[14] Branchiccela B., Castelli L., Corona M., Diaz-Cetti S., Invernizzi C., de la Escalera G.M., Mendoza Y., Santos E., Silva C., Zunino P. (2019). Impact of nutritional stress on the honeybee colony health. Sci. Rep..

[15] Di Pasquale G., Alaux C., Le Conte Y., Odoux J.F., Pioz M., Vaissiere B.E., Belzunces L.P., Decourtye A. (2016). Variations in the availability of pollen resources affect honey bee health. PLoS ONE.

[16] Di Pasquale G., Salignon M., Le Conte Y., Belzunces L.P., Decourtye A., Kretzschmar A., Suchail S., Brunet J.L., Alaux C. (2013). Influence of pollen nutrition on honey bee health: Do pollen quality and diversity matter?. PLoS ONE.

[17] Kaluza B.F., Wallace H.M., Heard T.A., Minden V., Klein A., Leonhardt S.D. (2018). Social bees are fitter in more biodiverse environments. Sci. Rep..

[18] Gill R.J., Raine N.E. (2014). Chronic impairment of bumblebee natural foraging behaviour induced by sublethal pesticide exposure. Funct. Ecol..

[19] Arce A.N., David T.I., Randall E.L., Rodrigues A.R., Colgan T.J., Wurm Y., Gill R.J. (2017). Impact of controlled neonicotinoid exposure on bumblebees in a realistic field setting. J. Appl. Ecol..

[20] Colin T., Meikle W.G., Wu X.B., Barron A.B. (2019). Traces of a neonicotinoid induce precocious foraging and reduce foraging performance in honey bees. Environ. Sci. Technol..

[21] Gill R.J., Ramos-Rodriguez O., Raine N.E. (2012). Combined pesticide exposure severely affects individual- and colony-level traits in bees. Nature.

[22] Lunardi S.J., Zaluski R., Orsi R.O. (2017). Evaluation of motor changes and toxicity of insecticides Fipronil and Imidacloprid in Africanized honey bees (Hymenoptera: Apidae). Sociobiology.

[23] Sivakoff F.S., Gardiner M.M. (2017). Soil lead contamination decreases bee visit duration at sunflowers. Urban Ecosyst..

[24] Siviter H., Koricheva J., Brown M.J.F., Leadbeater E. (2018). Quantifying the impact of pesticides on learning and memory in bees. J. Appl. Ecol..

[25] Stanley D.A., Russel A.L., Morrison S.J., Rogers C., Raine N.E. (2016). Investigating the impacts of field-realistic exposure to a neonicotinoid pesticide on bumblebee foraging, homing ability and colony growth. J. Appl. Ecol..

[26] Goulson D., Nicholls E. (2016). The canary in the coalmine; bee declines as an indicator of environmental health. Sci. Prog..

[27] Kennedy C.M., Lonsdorf E., Neel M.C., Williams N.M., Ricketts T.H., Winfree R., Bommarco R., Brittain C., Burley A.L., Cariveau D. (2013). A global quantitative synthesis of local and landscape effects on wild bee pollinators in agroecosystems. Ecol. Lett..

[28] Wilson E.O. (1971). The Insect Societies.

[29] Bryden J., Gill R.J., Mitton R.A.A., Raine N.E., Jansen V.A.A. (2013). Chronic sublethal stress causes bee colony failure. Ecol. Lett..

[30] Siviter H., Brown M.J.F., Leadbeater E. (2018). Sulfoxaflor exposure reduces bumblebee reproductive success. Nature.

[31] Bogdanov S. (2006). Contaminants of bee products. Apidologie.

[32] Nascimento A.S., Chambo E.D., Oliveria D.J., Andrade B.R., Bonsucesso J.S., Carvalho C.A.L. (2018). Honey from stingless bee as indicator of contamination with metals. Sociobiology.

[33] Nascimento N.O., Nalini H.A., Ataide F., Abreu A.T., Antonini Y. (2018). Pollen storage by stingless bees as an environmental marker for metal contamination: Spatial and temporal distribution of metal elements. Sociobiology.

[34] Sanchez-Bayo F., Goka K. (2014). Pesticide residues and bees—A risk assessment. PLoS ONE.

[35] Skaldina O., Peraniemi S., Sorvari J. (2018). Ants and their nests as indicators for industrial heavy metal contamination. Environ. Pollut..

[36] Urbini A., Sparvoli E., Turillazzi S. (2006). Social paper wasps as bioindicators: A preliminary research with Polistes dominulus (Hymenoptera Vespidae) as a trace metal accumulator. Chemosphere.

[37] Murcia Morales M., Goméz Ramos M.J., Parrilla Vasquez P., Díaz Galiano F.J., García Valverde M., Manuel Flores J., Fernández-Alba A.R. (2020). Distribution of chemical residues in the beehive compartments and their transfer to the honeybee brood. Sci. Total Environ..

[38] Traynor K.S., Pettis J.S., Tarpy D.R., Mullin C.A., Frazier J.L., Frazier M., vanEngeldorp D. (2016). In-hive pesticide exposome: Assessing risks to migratory honey bees from in-hive pesticide contamination in the Eastern United States. Sci. Rep..

[39] Ostiguy N., Eitzer B. (2014). Overwintered brood comb honey: Colony exposure to pesticide residues. J. Apic. Res..

[40] Chauzat M.P., Faucon J.P. (2007). Pesticide residues in beeswax samples collected from honey bee colonies (*Apis mellifera* l.) in France. Pest Manag. Sci..

[41] Hladun K.R., Di N., Liu T.X., Trumble J.T. (2016). Metal contaminant accumulation in the hive: Consequences for whole-colony health and brood production in the honey bee (Apis mellifera L.). Environ. Toxicol. Chem..

[42] Exley C., Rotheray E., Goulson D. (2015). Bumblebee pupae contain high levels of aluminium. PLoS ONE.

[43] Crall J.D., Switzer C.M., Oppenheimer R.L., Versypt A.N.F., Dey B., Brown A., Eyster M., Guerin C., Pierce N.E., Combes S.A. (2018). Neonicotinoid exposure disrupts bumblebee nest behavior, social networks, and thermoregulation. Science.

[44] Gathmann A., Tscharntke T. (2002). Foraging ranges of solitary bees. J. Anim. Ecol..

[45] Couvillon M.J., Schürch R., Ratnieks F.L.W. (2014). Dancing bees communicate a foraging preference for rural lands in high-level agri-environment schemes. Curr. Biol..

[46] Moron D., Grzes I.M., Skorka P., Szentgyorgyi H., Laskowski R., Potts S.G., Woyciechowski M. (2012). Abundance and diversity of wild bees along gradients of heavy metal pollution. J. Appl. Ecol..

[47] Moron D., Szentgyorgyi H., Skorka P., Potts S.G., Woyciechowski M. (2014). Survival, reproduction and population growth of the bee pollinator, *Osmia rufa* ( Hymenoptera: Megachilidae), along gradients of heavy metal pollution. Insect Conserv. Divers..

[48] Gradish A.E., van der Steen J., Scott-Dupree C.D., Cabrera A.R., Cutler C.G., Goulson D., Klein O., Lehmann D.M., Luckmann J., O’Neill B. (2019). Comparison of pesticide exposure in honey bees (Hymenoptera: Apidae) and bumble bees (Hymenoptera: Apidae): Implications for risk assessments. Environ. Entomol..

[49] Sgolastra F., Hinarejos S., Pitts-Singer T.L., Boyle N.K., Joseph T., Luckmann J., Raine N.E., Singh R., Williams N.M., Bosch J. (2019). Pesticide exposure assessment paradigm for solitary bees. Environ. Entomol..

[50] Cameron A.S., Sadd B.M. (2020). Global trends in bumble bee health. Annu. Rev. Entomol..

[51] Grozinger M.C., Flenniken M.L. (2019). Bee viruses: Ecology, pathogenicity, and impacts. Annu. Rev. Entomol..

[52] Schmid-Hempel P. (1998). Parasites in Social Insects. Monographs in Behavior and Ecology.

[53] Schmid-Hempel P., Stauffer H.P. (1998). Parasites and flower choice of bumblebees. Anim. Behav..

[54] Harrison F.J., Roberts S.P. (2000). Flight respiration and energetics. Annu. Rev. Physiol..

[55] Gonzalez-Varo J.P., Biesmeijer J.C., Bonmarco R., Potts S.G., Schweiger O., Smith H.G., Steffan-Dewenter I., Szentgyorgyi H., Woyciechowski M., Vila M. (2013). Combined effects of global change pressures on animal-mediated pollination. Trends Ecol. Evol..

[56] Goulson D., Lye G.C., Darvill B. (2008). Decline and conservation of bumble bees. Annu. Rev. Entomol..

[57] Vanbergen A.J., Baude M., Biesmeijer J.C., Britton N.F., Brown M.J.F., Brown M., Byden J., Budge G.E., Bull J.C., Carvell C. (2013). Threats to an ecosystem service: Pressures on pollinators. Front. Ecol. Environ..

[58] vanEngelsdorp D., Evans J.D., Seagerman C., Mullin C., Haubruge E., Nguyen B.K., Frazier M., Frazier J., Cox-Foster D., Chen Y.P. (2009). Colony Collapse Disorder: A descriptive study. PLoS ONE.

[59] David A., Botias C., Abdul-Sada A., Nicholls E., Rotheray E.L., Hill E.M., Goulson D. (2016). Widespread contamination of wildflower and bee-collected pollen with complex mixtures of neonicotinoids and fungicides commonly applied to crops. Environ. Int..

[60] Tosi S., Costa C., Vesco U., Quaglia G., Guido G. (2018). A 3-year survey of Italian honey bee-collected pollen reveals widespread contamination by agricultural pesticides. Sci. Total Environ..

[61] Botias C., David A., Hill E.M., Goulson D. (2017). Quantifying exposure of wild bumblebees to mixtures of agrochemicals in agricultural and urban landscapes. Environ. Pollut..

[62] Botias C., David A., Hill E.M., Goulson D. (2016). Contamination of wild plants near neonicotinoid seed-treated crops, and implications for non-target insects. Sci. Total Environ..

[63] Barganska Z., Slebioda M., Namiesnik J. (2016). Honey bees and their products: Bioindicators of environmental contamination. Crit. Rev. Environ. Sci. Technol..

[64] Bromenshenk J.J., Gudatis J.L., Carlson S.R., Thomas J.M., Simmons M.A. (1991). Population-dynamics of honey-bee nucleus colonies exposed to industrial pollutants. Apidologie.

[65] Conti E.M., Botre F. (2001). Honeybees and their products as potential bioindicators of heavy metals contamination. Environ. Monit. Assess..

[66] Costa A., Veca M., Barberis M., Tosti A., Notaro G., Nava S., Lazzari M., Agazzi A., Tangorra F.M. (2019). Heavy metals on honeybees indicate their concentration in the atmosphere. A proof of concept. Ital. J. Anim. Sci..

[67] Lazor P., Tomas J., Toth T., Toth J., Ceryova S. (2012). Monitoring of air pollution and atmospheric deposition of heavy metals by analysis of honey. J. Microbiol. Biotechnol. Food Sci..

[68] Perugini M., Manera M., Grotta L., Abete M.C., Tarasco R., Amorena M. (2011). Heavy metal (Hg, Cr, Cd, and Pb) contamination in urban areas and wildlife reserves: Honeybees as bioindicators. Biol. Trace Elem. Res..

[69] Skorbilowicz E., Skorbilowicz M., Ciesluk I. (2018). Bees as bioindicators of environmental pollution with metals in an urban area. J. Ecol. Eng..

[70] Smith K.E., Weis D., Amini M., Shiel A.E., Lai V.W.M., Gordon K. (2019). Honey as a biomonitor for a changing world. Nat. Sustain..

[71] Szentgyorgyi H., Blinow A., Eremeeva N., Luzyanin S., Grzes I.M., Woyciechowski M. (2011). Bumblebees (Bombidae) along pollution gradient—heavy metal accumulation, species diversity, and *Nosema bombi* infection level. Pol. J. Ecol..

[72] Dzugan M., Wesolowska M., Zagula G., Kaczmarski M., Czernicka M., Puchalski C. (2018). Honeybees (*Apis mellifera*) as a biological barrier for contamination of honey by environmental toxic metals. Environ. Monit. Assess..

[73] Gall J.E., Boyd R.S., Rajakaruna N. (2015). Transfer of heavy metals through terrestrial food webs: A review. Environ. Monit. Assess..

[74] Gutierrez M., Molero R., Gaju M., van der Steen J., Porrini C., Ruiz J.A. (2015). Assessment of heavy metal pollution in Cordoba (Spain) by biomonitoring foraging honeybee. Environ. Monit. Assess..

[75] Krakowska A., Muszynska B., Reczynski W., Opoka W., Turski W. (2015). Trace metal analyses in honey samples from selected countries. A Potential Use Bio-Monit. Int. J. Environ. Anal. Chem..

[76] Lawal O.A., Ademolu K.O., Aina S.A., Abiade A.N. (2014). Influence of nesting habitats on the gut enzymes activity and heavy metal composition of *Apis mellifera andersonii* L. (Hymenoptera: Apidae). Afr. Entomol..

[77] Matin G., Kargar N., Buyukisik H.B. (2016). Bio-monitoring of cadmium, lead, arsenic and mercury in industrial districts of Izmir, Turkey by using honey bees, propolis and pine tree leaves. Ecol. Eng..

[78] Silici S., Uluozlu O.D., Tuzen M., Soylak M. (2016). Honeybees and honey as monitors for heavy metal contamination near thermal power plants in Mugla, Turkey. Toxicol. Ind. Health.

[79] Yazgan S., Horn H., Isengard H.D. (2006). Honey as bio indicator by screening the heavy metal content of the environment. Dtsch. Lebensm.-Rundsch..

[80] Polidori C., Pastor A., Jorge A., Pertusa J. (2018). Ultrastructural alterations of midgut epithelium, but not greater wing fluctuating asymmetry, in paper wasps (*Polistes dominula*) from urban environments. Microsc. Microanal..

[81] McEachran A.D., Blackwell B.R., Hanson J.D., Wooten K.J., Mayer G.D., Cox S.B., Smith P.N. (2015). Antibiotics, bacteria, and antibiotic resistance genes: Aerial transport from cattle feed yards via particulate matter. Environ. Health Perspect..

[82] Tapparo A., Marton D., Giorio C., Zanella A., Solda L., Marzaro M., Vivan L., Girolami V. (2012). Assessment of the environmental exposure of honeybees to particulate matter containing neonicotinoid insecticides coming from corn coated seeds. Environ. Sci. Technol..

[83] Marzaro M., Vivan L., Targa A., Mazzon L., Mori N., Greatti M., Toffolo E.P., Di Bernardo A., Giorio C., Marton D. (2011). Lethal aerial powdering of honey bees with neonicotinoids from fragments of maize seed coat. Bull. Insectol..

[84] Zhou X.T., Taylor M.P., Davies P.J. (2018). Tracing natural and industrial contamination and lead isotopic compositions in an Australian native bee species. Environ. Pollut..

[85] Zhou X.T., Taylor M.P., Davies P.J., Prasad S. (2018). Identifying sources of environmental contamination in European honey bees (Apis mellifera) using trace elements and lead isotopic compositions. Environ. Sci. Technol..

[86] Bell J.N.B., Power S.A., Jarraud N., Agrawal M., Davies C. (2011). The effects of air pollution on urban ecosystems and agriculture. Int. J. Sustain. Dev. World Ecol..

[87] Lukowski A., Popek R., Jagiello R., Maderek E., Karolewski P. (2018). Particulate matter on two *Prunus* spp. decreases survival and performance of the folivorous beetle *Gonioctena quinquepunctata*. Environ. Sci. Pollut. Res..

[88] Negri I., Mavris C., Di Prisco G., Caprio E., Pellecchia M. (2015). Honey bees (*Apis mellifera*, L.) as active samplers of airborne particulate matter. PLoS ONE.

[89] Pellecchia M., Negri I. (2018). Particulate matter collection by honey bees (Apis mellifera, L.) near to a cement factory in Italy. Peerj.

[90] Tan Y.Q., Dion E., Monteiro A. (2018). Haze smoke impacts survival and development of butterflies. Sci. Rep..

[91] Vanderstock A.M., Latty T., Leonard R.J., Hochuli D.F. (2019). Mines over matter: Effects of foliar particulate matter on the herbivorous insect, Helicoverpa armigera. J. Appl. Entomol..

[92] Peterson E.M., Wooten K.J., Subbiah S., Anderson T.A., Longing S., Smith P.N. (2017). Agrochemical mixtures detected on wildflowers near cattle feed yards. Environ. Sci. Technol. Lett..

[93] Kreider M.L., Panko J.M., McAtee B.L., Sweet L.I., Finley B.L. (2010). Physical and chemical characterization of tire-related particles: Comparison of particles generated using different methodologies. Sci. Total Environ..

[94] Pant P., Baker S.J., Shukla A., Maikawa C., Pollitt K.J.G., Harrison R.M. (2015). The PM10 fraction of road dust in the UK and India: Characterization, source profiles and oxidative potential. Sci. Total Environ..

[95] Pant P., Harrison R.M. (2013). Estimation of the contribution of road traffic emissions to particulate matter concentrations from field measurements: A review. Atmos. Environ..

[96] Dominici F., Wang Y., Correia A.W., Ezzati M., Pope C.A., Dockery D.W. (2015). Chemical composition of fine particulate matter and life expectancy in 95 US counties between 2002 and 2007. Epidemiology.

[97] Kelly F.J., Fuller G.W., Walton H.A., Fussell J.C. (2012). Monitoring air pollution: Use of early warning systems for public health. Respirology.

[98] Kim K.H., Jahan S.A., Kabir E. (2013). A review on human health perspective of air pollution with respect to allergies and asthma. Environ. Int..

[99] Calatayud-Vernich P., Calatayud F., Simo E., Pico Y. (2018). Pesticide residues in honey bees, pollen and beeswax: Assessing beehive exposure. Environ. Pollut..

[100] Chauzat M.P., Martel A.C., Cougoule N., Porta P., Lachaize J., Zeggane S., Aubert M., Carpentier P., Faucon J.P. (2011). An assessment of honeybee colony matrices, *Apis mellifera* (Hymenoptera Apidae) to monitor pesticide presence in continental France. Environ. Toxicol. Chem..

[101] Daniele G., Giroud B., Jabot C., Vulliet E. (2018). Exposure assessment of honeybees through study of hive matrices: Analysis of selected pesticide residues in honeybees, beebread, and beeswax from French beehives by LC-MS/MS. Environ. Sci. Pollut. Res..

[102] Gonzalez-Martin M.I., Revilla I., Betances-Salcedo E.V., Vivar-Quintana A.M. (2018). Pesticide residues and heavy metals in commercially processed propolis. Microchem. J..

[103] Manning R. (2018). Chemical residues in beebread, honey, pollen and wax samples collected from bee hives placed on canola crops in Western Australia. J. Apic. Res..

[104] Porrini C., Caprio E., Tesoriero D., Di Prisco G. (2014). Using honey bee as bioindicator of chemicals in Campanian agroecosystems (South Italy). Bull. Insectol..

[105] Ravoet J., Reybroeck W., de Graaf D.C. (2015). Pesticides for apicultural and/or agricultural application found in Belgian honey bee wax combs. Bull. Environ. Contam. Toxicol..

[106] Valdovinos-Flores C., Alcantar-Rosales V.M., Gaspar-Ramirez O., Saldana-Loza L.M., Dorantes-Ugalde J.A. (2017). Agricultural pesticide residues in honey and wax combs from Southeastern, Central and Northeastern Mexico. J. Apic. Res..

[107] Kammoun S., Mulhauser B., Aebi A., Mitchell E.A.D., Glauser G. (2019). Ultra-trace level determination of neonicotinoids in honey as a tool for assessing environmental contamination. Environ. Pollut..

[108] Hoffmann B.D. (2011). Eradication of populations of an invasive ant in northern Australia: Successes, failures and lessons for management. Biodivers. Conserv..

[109] Buczkowski G., Wossler T.C. (2019). Controlling invasive Argentine ants, *Linepithema humile*, in conservation areas using horizontal insecticide transfer. Sci. Rep..

[110] Calibeo D., Oi F., Oi D., Mannion C. (2017). Insecticides for suppression of *Nylanderia fulva*. Insects.

[111] Sakamoto Y., Hayashi T.I., Inoue M.N., Ohnishi H., Kishimoto T., Goka K. (2019). Effects of fipronil on non-target ants and other invertebrates in a program for eradication of the Argentine ant, *Linepithema humile*. Sociobiology.

[112] Plentovich S., Swenson C., Reimer N., Richardson M., Garon N. (2010). The effects of hydramethylnon on the tropical fire ant, *Solenopsis geminata* (Hymenoptera: Formicidae), and non-target arthropods on Spit Island, Midway Atoll, Hawaii. J. Insect Conserv..

[113] Pisa L.W., Amaral-Rogers V., Belzunces L.P., Bonmatin J.M., Downs C.A., Goulson D., Kreutzweiser D.P., Krupke C., Liess M., McField M. (2015). Effects of neonicotinoids and fipronil on non-target invertebrates. Environ. Sci. Pollut. Res..

[114] Wood T.J., Goulson D. (2017). The environmental risks of neonicotinoid pesticides: A review of the evidence post 2013. Environ. Sci. Pollut. Res..

[115] Alburaki M., Chen D., Skinner J.A., Meikle W.G., Tarpy D.R., Adamczyk J., .Stewart S.D. (2018). Honey bee survival and pathogen prevalence: From the perspective of landscape and exposure to pesticides. Insects.

[116] Calatayud-Vernich P., Calatayud F., Simo E., Aguilar J.A.P., Pico Y. (2019). A two-year monitoring of pesticide hazard in-hive: High honey bee mortality rates during insecticide poisoning episodes in apiaries located near agricultural settings. Chemosphere.

[117] Calatayud-Vernich P., Calatayud F., Simo E., Suarez-Varela M.M., Pico Y. (2016). Influence of pesticide use in fruit orchards during blooming on honeybee mortality in 4 experimental apiaries. Sci. Total Environ..

[118] Tsvetkov N., Samson-Robert O., Sood K., Patel H.S., Malena D.A., Gajiwala P.H., Maciukiewicz P., Fournier V., Zayed A. (2017). Chronic exposure to neonicotinoids reduces honey bee health near corn crops. Science.

[119] Woodcock B.A., Bullock J.M., Shore R.F., Heard M.S., Pereira M.G., Redhead J., Ridding L., Dean H., Sleep D., Henrys P. (2017). Country-specific effects of neonicotinoid pesticides on honey bees and wild bees. Science.

[120] Woodcock B.A., Isaac N.J.B., Bullock J.M., Roy D.B., Garthwaite D.G., Crowe A., Pywell R.F. (2016). Impacts of neonicotinoid use on long-term population changes in wild bees in England. Nat. Commun..

[121] Aghamirlou H.M., Khadem M., Rahmani A., Sadeghian M., Mahvi A.H., Akbarzadeh A., Nazmara S. (2015). Heavy metals determination in honey samples using inductively coupled plasma-optical emission spectrometry. J. Environ. Health Sci. Eng..

[122] Altunatmaz S.S., Tarhan D., Aksu F., Barutcu U.B., Or M.E. (2017). Mineral element and heavy metal (cadmium, lead and arsenic) levels of bee pollen in Turkey. Food Sci. Technol..

[123] Bratu I., Georgescu C. (2005). The comparative study of heavy metals contamination of honey and flowers coming from a chemically polluted area. Rev. Chim..

[124] Celechovska O., Vorlova L. (2001). Groups of honey—Physicochemical properties and heavy metals. Acta Vet. Brno.

[125] Formicki G., Gren A., Stawarz R., Zysk B., Gal A. (2013). Metal content in honey, propolis, wax, and bee pollen and implications for metal pollution monitoring. Pol. J. Environ. Stud..

[126] Kovacik J., Gruz J., Biba O., Hedbavny J. (2016). Content of metals and metabolites in honey originated from the vicinity of industrial town Kosice (eastern Slovakia). Environ. Sci. Pollut. Res..

[127] Leita L., Muhlbachova G., Cesco S., Barabttini R., Mondini C. (1996). Investigation of the use of honey bees and honey bee products to assess heavy metals contamination. Environ. Monit. Assess..

[128] Ruschioni S., Riolo P., Minuz R.L., Stefano M., Cannella M., Porrini C., Isidoro N. (2013). Biomonitoring with honeybees of heavy metals and pesticides in nature reserves of the Marche region (Italy). Biol. Trace Elem. Res..

[129] Sitarz-Palczak E., Kalembkiewicz J., Galas D. (2015). Evaluation of the content of selected heavy metals in samples of Polish honeys. J. Ecol. Eng..

[130] Spiric D., Ciric J., Dordevic V., Nikolic D., Jankovic S., Nikolic A., Petrovic Z., Katanic N., Teodorovic V. (2019). Toxic and essential element concentrations in different honey types. Int. J. Environ. Anal. Chem..

[131] Burden C.M., Elmore C., Hladun K.R., Trumble J.T., Smith B.H. (2016). Acute exposure to selenium disrupts associative conditioning and long-term memory recall in honey bees (*Apis mellifera*). Ecotoxicol. Environ. Saf..

[132] Burden C.M., Morgan M.O., Hladun K.R., Amdam G.V., Trumble J.T., Smith B.H. (2019). Acute sublethal exposure to toxic heavy metals alters honey bee (Apis mellifera) feeding behavior. Sci. Rep..

[133] Eeva T., Sorvari J., Kolvunen V. (2004). Effects of heavy metal pollution on red wood ant (*Formica* s. *str.)* populations. Environ. Pollut..

[134] Nisbet C., Guler A., Biyik S. (2019). Effects of different environmental conditions on the cognitive function of honeybee (*Apis mellifera* L.) and mineral content of honey. Ank. Univ. Vet. Fak. Derg..

[135] Sgolastra F., Blasioli S., Renzi T., Tosi S., Medrzycki P., Molowny-Horas R., Porrini C., Braschi I. (2018). Lethal effects of Cr(III) alone and in combination with propiconazole and clothianidin in honey bees. Chemosphere.

[136] Skaldina O., Sorvari J., Kesari K.K. (2009). Ecotoxicological Effects of Heavy Metal Pollution on Economically Important Terrestrial Insects, in Networking of Mutagens in Environmental Toxicology.

[137] Sorvari J., Eeva T. (2010). Pollution diminishes intra-specific aggressiveness between wood ant colonies. Sci. Total Environ..

[138] Sorvari J., Rantala L.M., Rantala M.J., Hakkarainen H., Eeva T. (2007). Heavy metal pollution disturbs immune response in wild ant populations. Environ. Pollut..

[139] Szentgyorgyi H., Moron D., Nawrocka A., Tofilski A., Woyciechowski M. (2017). Forewing structure of the solitary bee *Osmia bicornis* developing on heavy metal pollution gradient. Ecotoxicology.

[140] Kampa M., Castanas E. (2008). Human health effects of air pollution. Environ. Pollut..

[141] Järup L. (2003). Hazards of heavy metal contamination. Br. Med. Bull..

[142] Tragust S., Tartally A., Espadaler X., Billen J. (2016). Histopathology of Laboulbeniales (Ascomycota: Laboulbeniales): Ectoparasitic fungi on ants (Hymenoptera: Formicidae). Myrmecol. News.

[143] Tragust S., Feldhaar H., Espadaler X., Pedersen J.S. (2015). Rapid increase of the parasitic fungus *Laboulbenia formicarum* in supercolonies of the invasive garden ant *Lasius neglectus*. Biol. Invasions.

[144] Bos N., Kankaanpaa-Kukkonen V., Freitak D., Stucki D., Sundstrom L. (2019). Comparison of twelve ant species and their susceptibility to fungal infection. Insects.

[145] Valles S.M., Rivers A.R. (2019). Nine new RNA viruses associated with the fire ant *Solenopsis invicta* from its native range. Virus Genes.

[146] Li J.L., Qin H.R., Wu J., Sadd B.M., Wang X.H., Evans J.D., Peng W.J., Chen Y.P. (2012). The prevalence of parasites and pathogens in Asian honeybees *Apis cerana* in China. PLoS ONE.

[147] Manfredini F., Shoemaker D., Grozinger C.M. (2016). Dynamic changes in host-virus interactions associated with colony founding and social environment in fire ant queens (*Solenopsis invicta*). Ecol. Evol..

[148] Porter S.D., Valles S.M., Oi D.H. (2013). Host specificity and colony impacts of the fire ant pathogen, Solenopsis invicta virus 3. J. Invertebr. Pathol..

[149] Tufts D.M., Hunter W.B., Bextine B. (2010). Discovery and effects of Texas Solenopsis invicta virus [SINV-1 (TX5)] on red imported fire ant populations. J. Invertebr. Pathol..

[150] Valles S.M., Porter S.D. (2015). Dose response of red imported fire ant colonies to Solenopsis invicta virus 3. Arch. Virol..

[151] Valles S.M. (2012). Positive-strand RNA viruses infecting the red imported fire ant, *Solenopsis invicta*. Psyche.

[152] Cordes N., Huang W.F., Strange J.P., Cameron S.A., Griswold T.L., Lozier J.D., Solter L.F. (2012). Interspecific geographic distribution and variation of the pathogens *Nosema bombi* and *Crithidia* species in United States bumble bee populations. J. Invertebr. Pathol..

[153] Brown M.J.F., Loosli R., Schmid-Hempel P. (2000). Condition-dependent expression of virulence in a trypanosome infecting bumblebees. Oikos.

[154] Brown M.J.F., Schmid-Hempel R., Schmid-Hempel P. (2003). Strong context-dependent virulence in a host-parasite system: Reconciling genetic evidence with theory. J. Anim. Ecol..

[155] Fauser A., Sandrock C., Neumann P., Sadd B. (2017). Neonicotinoids override a parasite exposure impact onhibernation success of a key bumblebee pollinator. Ecol. Entomol..

[156] Genersch E. (2010). Honey bee pathology: Current threats to honey bees and beekeeping. Appl. Microbiol. Biotechnol..

[157] Goblirsch M. (2018). Nosema ceranae disease of the honey bee (Apis mellifera). Apidologie.

[158] Paris L., El Alaoui H., Delbac F., Diogon M. (2018). Effects of the gut parasite *Nosema ceranae* on honey bee physiology and behavior. Curr. Opin. Insect Sci..

[159] Lemaitre B., Hoffmann J. (2007). The host defense of *Drosophila melanogaster*. Annu. Rev. Immunol..

[160] Otti O., Tragust S., Feldhaar H. (2014). Unifying external and internal immune defences. Trends Ecol. Evol..

[161] Cremer S., Armitage S.A.O., Schmid-Hempel P. (2007). Social immunity. Curr. Biol..

[162] Broderick N.A., Welchman D.P., Lemaitre B., Rolff J., Reynolds S.E. (2009). Recognition and Response to Microbial Infection in Drosophila, in Insect Infection and Immunity.

[163] Wilson-Rich N., Bonoan R.E., Taylor E., Lwanga L., Starks P.T. (2019). An improved method for testing invertebrate encapsulation response as shown in the honey bee. Insectes Soc..

[164] Allander K., Schmid-Hempel P. (2000). Immune defence reaction in bumble-bee workers after a previous challenge and parasitic coinfection. Funct. Ecol..

[165] Sorvari J., Hakkarainen H., Rantala M.J. (2008). Immune defense of ants is associated with changes in habitat characteristics. Environ. Entomol..

[166] Li X.C., Schuler M.A., Berenbaum M.R. (2007). Molecular mechanisms of metabolic resistance to synthetic and natural xenobiotics. Annu. Rev. Entomol..

[167] Berenbaum M.R., Johnson R.M. (2015). Xenobiotic detoxification pathways in honey bees. Curr. Opin. Insect Sci..

[168] Collison E.J., Hird H., Tyler C.R., Cresswell J.E. (2018). Effects of neonicotinoid exposure on molecular and physiological indicators of honey bee immunocompetence. Apidologie.

[169] Alaux C., Brunet J.L., Dussaubat C., Mondet F., Tchamitchan S., Cousin M., Brillard J., Baldy A., Belzunces L.P., Le Conte Y. (2010). Interactions between *Nosema* microspores and a neonicotinoid weaken honeybees (*Apis*
*mellifera*). Environ. Microbiol..

[170] Reeves A.M., O’Neal S.T., Fell R.D., Brewster C.C., Anderson T.D. (2018). In-hive acaricides alter biochemical and morphological indicators of honey bee nutrition, immunity, and development. J. Insect Sci..

[171] O’Neal S.T., Reeves A.M., Fell R.D., Brewster C.C., Anderson T.D. (2019). Chlorothalonil exposure alters virus susceptibility and markers of immunity, nutrition, and development in honey bees. J. Insect Sci..

[172] Paris L., Roussel M., Pereira B., Delbac F., Diogon M. (2017). Disruption of oxidative balance in the gut of the western honeybee *Apis mellifera* exposed to the intracellular parasite *Nosema ceranae* and to the insecticide fipronil. Microb. Biotechnol..

[173] Carneiro L.S., Martinez L.C., Goncalves W.G., Santana L.M., Serraeo J.E. (2020). The fungicide iprodione affects midgut cells of non-target honey bee *Apis mellifera* workers. Ecotoxicol. Environ. Saf..

[174] Lämsä J., Kuusela E., Tuomi J., Juntunen S., Watts P.C. (2018). Low dose of neonicotinoid insecticide reduces foraging motivation of bumblebees. Proc. R. Soc. B-Biol. Sci..

[175] Moret Y., Schmid-Hempel P. (2000). Survival for immunity: The price of immune system activation for bumblebee workers. Science.

[176] Ardia D.R., Gantz J.E., Schneider B.C., Strebel S. (2012). Costs of immunity in insects: An induced immune response increases metabolic rate and decreases antimicrobial activity. Funct. Ecol..

[177] Klein S., Cabirol A., Devaud J.M., Barron A.B., Lihoreau M. (2017). Why bees are so vulnerable to environmental stressors. Trends Ecol. Evol..

[178] Boucias D.G., Stokes C., Storey G., Pendland J.C. (1996). The effects of imidacloprid on the termite *Reticulitermes flavipes* and its interaction with the mycopathogen Beauveria bassiana. Pflanzenschutz-Nachr. Bayer.

[179] Raymann K., Moran N.A. (2018). The role of the gut microbiome in health and disease of adult honey bee workers. Curr. Opin. Insect Sci..

[180] Engel P., Kwong W.K., McFrederick Q., Anderson K.E., Barribeau S.M., Chandler J.A., Cornman R.S., Dainat J., de Miranda J.R., Doublet V. (2016). The bee microbiome: Impact on bee health and model for evolution and ecology of host-microbe interactions. Mbio.

[181] Hamdi C., Balloi A., Essanaa J., Crotti E., Gonella E., Raddadi N., Ricci I., Boudabous A., Borin S., Manino A. (2011). Gut microbiome dysbiosis and honeybee health. J. Appl. Entomol..

[182] Rothman J.A., Leger L., Kirkwood J.S., McFrederick Q.S. (2019). Cadmium and selenate exposure affects the honey bee microbiome and metabolome, and bee-associated bacteria show potential for bioaccumulation. Appl. Environ. Microbiol..

[183] Yang Y., Ma S.L., Yan Z.X., Liu F., Diao Q.Y., Dai P.L. (2019). Effects of three common pesticides on survival, food consumption and midgut bacterial communities of adult workers *Apis cerana* and *Apis mellifera*. Environ. Pollut..

[184] Motta E.V.S., Raymann K., Moran N.A. (2018). Glyphosate perturbs the gut microbiota of honey bees. Proc. Natl. Acad. Sci. USA.

[185] Kakumanu M.L., Reeves A.M., Anderson T.D., Rodrigues R.R., Williams M.A. (2016). Honey bee gut microbiome is altered by in-hive pesticide exposures. Front. Microbiol..

[186] Blot N., Veillat L., Rouze R., Delatte H. (2019). Glyphosate, but not its metabolite AMPA, alters the honeybee gut microbiota. PLoS ONE.

[187] Lucky A. (2009). Urban ants of North America and Europe: Identification, biology, and management. Syst. Entomol..

[188] Lutinski J.A., Lopes B.C., de Morais A.B.B. (2013). Urban ant diversity (Hymenoptera: Formicidae) in ten cities of southern Brazil. Biota Neotrop..

[189] Savage A.M., Hackett B., Guenard B., Youngsteadt E.K., Dunn R.R. (2015). Fine-scale heterogeneity across Manhattan’s urban habitat mosaic is associated with variation in ant composition and richness. Insect Conserv. Divers..

[190] Youngsteadt E., Appler R.H., Lopez-Uribe M.M., Tarpy D.R., Frank S.D. (2015). Urbanization increases pathogen pressure on feral and managed honey bees. PLoS ONE.

[191] Johnson R.M. (2015). Honey bee toxicology. Annu. Rev. Entomol..

[192] Fairbrother A., Purdy J., Anderson T., Fell R. (2014). Risks of neonicotinoid insecticides to honeybees. Environ. Toxicol. Chem..

[193] Lopez D.R., Ahumada D.A., Diaz A.C., Guerrero J.A. (2014). Evaluation of pesticide residues in honey from different geographic regions of Colombia. Food Control.

[194] Luken D.J., von der Ohe W. (2018). A research about different residues in pollen and honey samples. Hazards Pestic. Bees.

[195] Mullin C.A., Frazier M., Frazier J.L., Ashcraft S., Simonds R., vanEngelsdorp D., Pettis J.S. (2010). High levels of miticides and agrochemicals in North American apiaries: Implications for honey bee health. PLoS ONE.

[196] Stanley D.A., KSmith E., Raine N.E. (2015). Bumblebee learning and memory is impaired by chronic exposure to a neonicotinoid pesticide. Sci. Rep..

[197] Baron G.L., Raine N.E., Brown M.J.F. (2017). General and species-specific impacts of a neonicotinoid insecticide on the ovary development and feeding of wild bumblebee queens. Proc. R. Soc. B-Biol. Sci..

[198] Brandt A., Gorenflo A., Siede R., Meixner M., Büchler R. (2016). The neonicotinoids thiacloprid, imidacloprid, and clothianidin affect the immunocompetence of honey bees (*Apis mellifera* L.). J. Insect Physiol..

[199] Brandt A., Grikscheidt K., Siede R., Grosse R., Meixner M.D., Büchler R. (2017). Immunosuppression in honeybee queens by the neonicotinoids thiacloprid and clothianidin. Sci. Rep..

[200] Gauthier M., Aras P., Paquin J., Boily M. (2018). Chronic exposure to imidacloprid or thiamethoxam neonicotinoid causes oxidative damages and alters carotenoid-retinoid levels in caged honey bees (*Apis mellifera*). Sci. Rep..

[201] Tesovnik T., Zorc M., Ristanic M., Glavinic U., Stevanovic J., Narat M., Stanimirovic Z. (2020). Exposure of honey bee larvae to thiamethoxam and its interaction with Nosema ceranae infection in adult honey bees. Environ. Pollut..

[202] Tesovnik T., Cizelj I., Zorc M., Citar M., Bozic J., Glavan G., Narat M. (2017). Immune related gene expression in worker honey bee (Apis mellifera carnica) pupae exposed to neonicotinoid thiamethoxam and Varroa mites (Varroa destructor). PLoS ONE.

[203] Aufauvre J., Misme-Aucouturier B., Vigues B., Texier C., Delbac F., Blot N. (2014). Transcriptome analyses of the honeybee response to Nosema ceranae and insecticides. PLoS ONE.

[204] McMenamin A.J., Brutscher L.M., Glenny W., Flenniken M.L. (2016). Abiotic and biotic factors affecting the replication and pathogenicity of bee viruses. Curr. Opin. Insect Sci..

[205] Vidau C., Diogon M., Aufauvre J., Fontbonne R., Vigues B., Brunet J.L., Texier C., Biron D.G., Blot N., El Alaoui H. (2011). Exposure to sublethal doses of fipronil and thiacloprid highly increases mortality of honeybees previously infected by Nosema ceranae. PLoS ONE.

[206] Kairo G., Biron D.G., Ben Abdelkader F., Bonnet M., Tchamitchian S., Cousin M., Dussaubat C., Benoit B., Kretzschmar A., Belzunces L.P. (2017). *Nosema**ceranae*, fipronil and their combination compromise honey bee reproduction via changes in male physiology. Sci. Rep..

[207] Dussaubat C., Maisonnasse A., Crauser D., Tchamitchian S., Bonnet M., Cousin M., Kretzschmar A., Brunet J.L., Le Conte Y. (2016). Combined neonicotinoid pesticide and parasite stress alter honeybee queens’ physiology and survival. Sci. Rep..

[208] Coulon M., Schurr F., Martel A.C., Cougoule N., Begaud A., Mangoni P., Di Prisco G., Dalmon A., Alaux C., Ribiere-Chabert M. (2019). Influence of chronic exposure to thiamethoxam and chronic bee paralysis virus on winter honey bees. PLoS ONE.

[209] Straub L., Williams G.R., Vidondo B., Khongphinitbunjong K., Retschnig G., Schneeberger A., Chantawannakul P., Dietemann V., Neumann P. (2019). Neonicotinoids and ectoparasitic mites synergistically impact honeybees. Sci. Rep..

[210] Giglio A., Ammendola A., Battistella S., Naccarato A., Pallavicini A., Simeon E., Tagarelli A., Giulianini P.G. (2017). *Apis mellifera ligustica*, Spinola 1806 as bioindicator for detecting environmental contamination: A preliminary study of heavy metal pollution in Trieste, Italy. Environ. Sci. Pollut. Res..

[211] Raes H., Cornelis R., Rzeznik U. (1992). Distribution, accumulation and depuration of administered lead in adult honeybees. Sci. Total Environ..

[212] Polykretis P., Delfino G., Petrocelli I., Cervo R., Tanteri G., Montori G., Perito B., Branca J.J.V., Morucci G., Gulisano M. (2016). Evidence of immunocompetence reduction induced by cadmium exposure in honey bees (*Apis mellifera*). Environ. Pollut..

[213] Dabour K., Al Naggar Y., Masry S., Naiem E., Giesy J.P. (2019). Cellular alterations in midgut cells of honey bee workers (*Apis millefera* L.) exposed to sublethal concentrations of CdO or PbO nanoparticles or their binary mixture. Sci. Total Environ..

[214] Bové H., Bongaerts E., Slenders E., Bijnens E.M., Saenen N.D., Gyselaers W., Van Eyken P., Plusquin M., Roeffaers M.B.J., Ameloot M. (2019). Ambient black carbon particles reach the fetal side of human placenta. Nat. Commun..

[215] Kelly F.J., Fussell J.C. (2015). Linking ambient particulate matter pollution effects with oxidative biology and immune responses. Cell. Environ. Stressors Biol. Med..

[216] Kim K.H., Kabir E., Kabir S. (2015). A review on the human health impact of airborne particulate matter. Environ. Int..

[217] Liang C.S., Duan F.K., He K.B., Ma Y.L. (2016). Review on recent progress in observations, source identifications and countermeasures of PM2.5. Environ. Int..

[218] Kelly F.J., Fussell J.C. (2012). Size, source and chemical composition as determinants of toxicity attributable to ambient particulate matter. Atmos. Environ..

[219] Zaric N.M., Deljanin I., Ilijevic K., Stanisavljevic L., Ristic M., Grzetic I. (2018). Honeybees as sentinels of lead pollution: Spatio-temporal variations and source appointment using stable isotopes and Kohonen self-organizing maps. Sci. Total Environ..

[220] Sheldon M., Pinion C., Klyza J., Zimeri A.M. (2019). Pesticide contamination in central Kentucky urban honey: A pilot study. J. Environ. Health.

